# Evidence of population genetic structure in Ecuadorian Andean bears

**DOI:** 10.1038/s41598-024-53003-5

**Published:** 2024-02-03

**Authors:** Dario F. Cueva, Rebecca Zug, María José Pozo, Santiago Molina, Rodrigo Cisneros, Martín R. Bustamante, María de Lourdes Torres

**Affiliations:** 1 Laboratorio de Biotecnología Vegetal, Universidad San Francisco de Quito USFQ, Diego de Robles y Via Interoceanica s/n, Quito, 170157 Ecuador; 2 Laboratorio de Carnívoros, Universidad San Francisco de Quito USFQ, Diego de Robles y Vía Interoceanica s/n, Quito, 170157 Ecuador; 3 Fundación Zoológica del Ecuador, Pircapamaba s/n y Rumichupa, Guayllabamba, Quito Ecuador; 4 Departamento de Ciencias Biológicas y Agropecuarias, Universidad Técnica Particular de Loja, San Cayetano Alto, C/París s/n., 1101608 Loja, Ecuador

**Keywords:** Structural variation, Conservation biology

## Abstract

Wildlife conservation in Andean countries is a global priority because of the high levels of biodiversity and endemism. Historically, these countries have had limited resources to monitor wildlife (e.g., through genetic tools) and establish conservation programs. Focusing on the study and emblematic use of a few charismatic species has been a strategic approach to direct efforts for conservation and development planning. Consequently, the Andean bear is a flagship and umbrella species for highly biodiverse Andean countries like Ecuador. The few studies exploring the population genetics of this species have concluded that it has low genetic diversity and few units for conservation as populations appear to be well connected. However, these results might be attributed to ascertainment bias as studies have been performed with heterologous molecular markers. Here, using both mtDNA sequences and species-specific microsatellite markers, we show that Andean bears in Ecuador have population structure. Additionally, we found through the study of three Ecuadorian populations that the species might have a higher genetic diversity than we previously thought. These results could support the revision of research priorities, conservation, and planning strategies to improve connectivity for this species which occurs in crucial biodiversity hotspots.

## Introduction

Analysis of genetic variation using molecular markers is an important tool for the conservation of many species of wildlife^1,2^. In recent decades these techniques have become widely accepted, and the increase in affordability and publication of methods have made these tools more accessible to ecologists and conservation biologists^3–6^. Among other data, genetic analysis can provide information on distinct populations, connectivity between them, and breeding patterns^7–9^. These factors can be particularly important when identifying and conserving small or isolated populations with a higher risk of inbreeding depression, genetic drift, and thus an overall reduced fitness^10^. Issues related to small population size are of significant concern for large carnivores, which typically have far-ranging behavior and require extensive habitat connectivity to maintain viable populations^11^. Large carnivores naturally occur at lower densities but have a disproportionately strong influence on ecosystems, and their loss can trigger cascading effects that alter ecosystem function and can influence human health and food security^12–14^. As such, they are often used as proxies for the conservation of other species, ecosystems, and landscapes^15,16^.

Andean bears (*Tremarctos ornatus*) are endemic to the Tropical Andes and are distributed across narrow mountain corridors, where difficult topography and human settlements might prevent gene flow between populations. They are the largest carnivores in the Andes and play an important ecological role as seed dispersers^17^. Andean bears usually occur at elevations above 1000 m in a variety of highly biodiverse and unique Andean ecosystems (e.g., paramo, montane forests) that provide services, such as freshwater, to millions of people^17,18^. Their global conservation status is Vulnerable, with declining populations in the five Andean countries where they are distributed^19^. In Ecuador, Andean bears are listed as Endangered^20^, where an estimated 69% of the population lives outside protected areas^21^, often on unprotected private lands. The advancing agricultural frontier causes habitat loss and fragmentation and increases the opportunity for human-bear conflict when bears raid crops (e.g. corn) or kill livestock^22^. The aforementioned topics are considered in the Ecuadorian National Plan for the Conservation of the Andean Bear, which addresses research priorities^23^. Ecuadorian law prohibits hunting this species^24^, but landowners sometimes respond lethally^23^, adding human-caused mortality as a threat to potentially small populations. Human-wildlife conflict can be a form of habitat fragmentation when wildlife occurs in a hazardous matrix of human activity and poverty.

Understanding the genetic variation across the Andean bear distribution in Ecuador will provide important information on connectivity and can target conservation efforts toward the most vulnerable populations. As a flagship and umbrella species, improvements for the conservation of Andean bears can have far-reaching impacts on local biodiversity^17^. Compared to other ursids, the Andean bear is an understudied species, considering the lack of studies that report genetic analysis using species-specific markers^25^. Little is known about the use of specific genetic markers to understand the genetic diversity, population structure, management units, and conservation status of this important species. Previous studies have largely focused on the analysis of up to six mitochondrial DNA genes, which contain genetic information from maternal lineages, and a set of four to nine nuclear heterologous microsatellite (a.k.a. short sequence repeats—SSR) markers originally developed for *Ursus americanus* and other Carnivora species^25–30^. Recently, it has been suggested that the current set of microsatellite markers is inadequate for population inferences due to ascertainment bias which results in an underestimation of genetic diversity. This is explained by the fact that lower genetic variation is usually detected in the species for which the markers were not originally developed^25,31^. Furthermore, these heterologous markers include only di-nucleotide motif microsatellites, which impact allele score accuracy due to smeared patterns of peaks resulting from DNA polymerase slippage or cross recombination during repeated amplification cycles^32,33^. This can introduce genotyping errors, which further impact genetic diversity and structure estimations. Therefore, the ecological interpretation of previous works and the implications for conservation and management may require reconsideration due to bias attributed to marker choice^25,31^. Until now, no microsatellite markers have been developed specifically for Andean bear studies. Using species-specific markers is necessary to understand whether the low genetic diversity reported for this species is indeed attributed to ascertainment bias^25,31^. Here we describe the development and use of microsatellite markers for this species and the analysis of mitochondrial D-loop and COXII sequences to evaluate the genetic diversity and structure of Ecuadorian Andean bear populations. We discuss the implications of our results for the conservation of Andean bears in Ecuador and the highly biodiverse ecosystems where they live.

## Results

### Mitochondrial D-loop hyper-variable region 1 amplification, genetic diversity, and haplotype analysis

**Figure 1:**
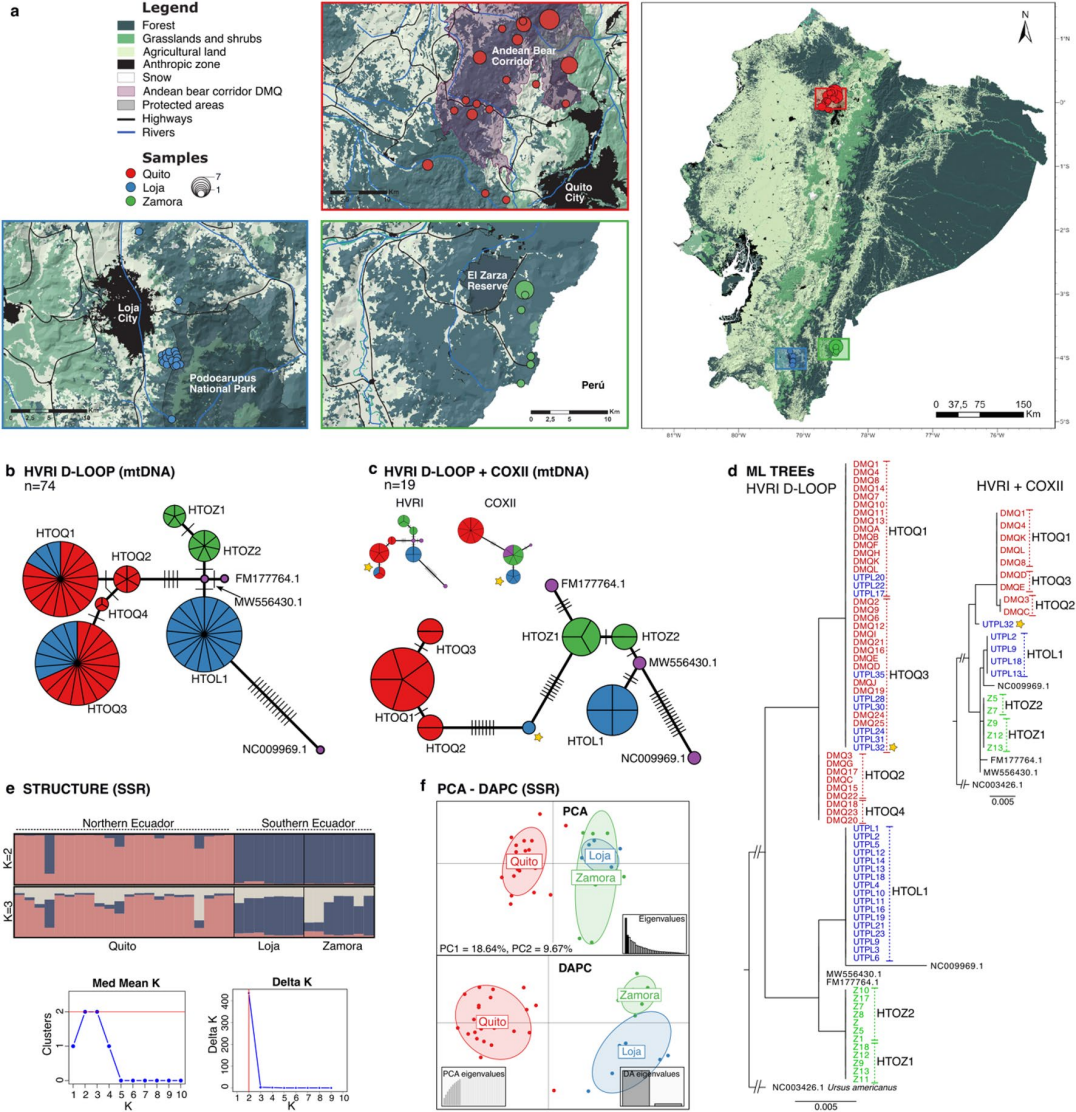
Sampling areas and genetic differentiation analyses in Ecuadorian Andean bears. (**a**) Map of sample collection sites in Quito (in red), Loja (in blue), and Zamora Chinchipe (in green). Land use layers are displayed using ESRI ArcGis Pro v.10.8.2 (source: http://ide.ambiente.gob.ec:8080/mapainteractivo/). (**b**) Haplotype network inferred from the analysis of the hypervariable region 1 of the D-loop in the mitochondrial DNA in 74 samples. The sequences available in the NCBI nucleotide database FM177764.1, MW556430.1 and NC009969.1 were incorporated. (**c**) Haplotype network from the analysis of the COXII gene + HVR1 sequences in 19 samples. Individual networks of the COXII gene and the HVRI are displayed for comparison. The yellow star represents the sample UTPL32 and how it segregates from the northern haplotypes when adding COXII data. (**d**) Phylogenetic relationships among individuals using mtDNA sequences from a Maximum-Likelihood optimization methodology with a Kimura-2 parameter substitution model. (**e**) Structure analysis from SSR data for 2 and 3 genetic clusters (K) as predicted as the best number of clusters to explain genetic variability in the Structure selector. (**f**) Principal Coordinates Analysis PCA and Discriminant Analysis of Principal Components DAPC from SSR data. PCA represents 28.31% of the variability, while the DAPC displays 71.46% of the total variability comprised in the first 10 components. Samples from Quito appear to be separated from samples from southern Ecuador (Loja and Zamora Chinchipe provinces). Further differences are observed between Loja and Zamora when analyzing more variability as displayed in the DAPC.

Our analysis included a total of 74 samples, which comprised 36 samples from a preliminary study in the Quito Metropolitan District in northern Ecuador^34^, and 38 new samples from southern Ecuador (Fig. 1a). All the new samples were amplified successfully, and sequence analysis was carried out on a 462 bp fragment from the hypervariable region 1 (HVR1) of the D-loop in the mitochondrial DNA. Only 7 haplotypes were identified across all sampling localities with a low degree of differentiation among them (π = 0.0077 ± 0.0044). Four haplotypes were found in the Quito Metropolitan District (n = 36), three in Loja (n = 26), and two in Zamora Chinchipe (n = 12) Provinces (Table 1, Fig. 1b). We found a moderate haplotype diversity (Hd = 0.82 ± 0.02) for Ecuador. The southern populations of Loja (Hd = 0.53 ± 0.09) and Zamora Chinchipe provinces (Hd = 0.53 ± 0.07) displayed lower genetic diversity than what was previously reported for Quito (Hd = 0.70 ± 0.04)^34^.

**Table 1:** Genetic diversity indexes obtained from the mitochondrial HVR1 analysis of the whole dataset.

mtDNA region	Population	N	S	H	*H*_*d*_ ± sd	π ± sd	Tajima’s D	Fu’s FS
HVRI D-loop 462 bp	Overall	74	8	7	0.82 ± 0.02	0.0077 ± 0.0044	2.80	3.54
	Quito	36	2	4	0.70 ± 0.04	0.0019 ± 0.0015	0.89	0.29
	Loja	26	7	3	0.53 ± 0.09	0.0069 ± 0.0041	2.35	5.91
	Zamora	12	1	2	0.53 ± 0.07	0.0011 ± 0.0011	0.00	1.15

N number of samples, S segregating sites, H number of haplotypes, *H*_*d*_ haplotype diversity, π nucleotide diversity, sd standard deviation. No statistical significance was found in any Tajima’s D or Fu’s Fs values.

The new haplotype from Loja Province (HTOL1: NCBI nucleotide database accession number MT425202.1) and the two new haplotypes from Zamora Chinchipe Province (HTOZ1: accession number MZ191063.1 and HTOZ2: accession number MZ191064.1) were exclusive to these localities (Fig. 1b). No shared haplotypes were found between Quito and Zamora Province. We only found 2 (HTOQ1 and HTOQ3) of the 4 previously reported haplotypes for Quito (HTOQ1-4, accession numbers: KX812512.1-KX812515.1)^34^ in individuals from Loja Province (although in low frequency) which are located hundreds of kilometers apart north to south. Yet, we did not find any shared haplotype between the Loja and Zamora Chinchipe populations, which are significantly closer but separated east to west by the mountainous terrain of the Andes (Fig. 1a,b,d). Similarly, the phylogenetic analysis shows that samples from Loja and Zamora are indeed distinct. All Zamora samples have a monophyletic origin, mirroring the monophyletic origin observed in Quito samples. In contrast, individuals from Loja have a parafiletic origin (Fig. 1d).

The analysis of molecular variance AMOVA revealed a high genetic variability (34.8%) among the populations. These results suggest the possibility of population structure. Finally, Tajima's D-neutrality and Fu's FS tests (Table 1) show that there have been no recent demographic changes in the population, such as expansions or bottlenecks (P > 0.05), although the low number of polymorphisms found in the sequences limits the application and interpretation of these tests.

### Mitochondrial COXII amplification, genetic diversity, and haplotype analysis

We selected a subset of 19 samples from our three study populations to test whether adding a new mitochondrial DNA fragment to the analysis would reveal further differences between the haplotypes initially found. These selected samples had good quantity and quality DNA and represented almost all haplotypes found with the HVR1 analysis. The amplified fragment corresponded to a 1082 bp product (Fig. S1) encompassing the entire COXII gene along with small segments from the flanking COXI and ATP8 genes. After trimming sequences for equal length, the data analysis was performed on a 972 bp region.

Our findings from the COXII gene analysis revealed just one haplotype attributed to each population (Fig. 1c), resulting in zero haplotype and nucleotide diversity within the populations (Table 2). Consequently, the AMOVA analysis of this region alone shows that 100% of the variance is solely attributed to population differentiation, reflecting a clear population genetic structure. Additionally, these COXII sequences (NCBI Accessions OR999075-OR999077) exhibited a significantly lower genetic diversity for this dataset of Ecuadorian samples (Hd 0.67) when compared to the HVR1 (Hd 0.85).

**Table 2:** Genetic diversity indexes obtained from the mitochondrial HVR1 and COXII gene analysis of a reduced dataset.

mtDNA region	Population	N	S	H	*H*_*d*_ ± sd	π ± sd	Tajima’s D	Fu’s FS
HVRI D-loop 462 bp	Overall	19	8	6	0.85 ± 0.04	0.0083 ± 0.0048	2.29	1.56
	Quito	9	2	3	0.67 ± 0.13	0.0017 ± 0.0015	0.16	− 0.10
	Loja	5	7	2	0.40 ± 0.23	0.0060 ± 0.0045	− 1.14	3.36
	Zamora	5	1	2	0.60 ± 0.17	0.0013 ± 0.0014	0.00	0.62
COXII 972 bp	Overall	19	7	3	0.67 ± 0.06	0.0037 ± 0.0021	2.60	5.58
	Quito	9	0	1	0.00 ± 0.00	0.0000 ± 0.0000	0.00	0.00
	Loja	5	0	1	0.00 ± 0.00	0.0000 ± 0.0000	0.00	0.00
	Zamora	5	0	1	0.00 ± 0.00	0.0000 ± 0.0000	0.00	0.00
HVRI–COXII 1434 bp	Overall	19	15	7	0.87 ± 0.04	0.0052 ± 0.0028	2.72	3.00
	Quito	9	2	3	0.67 ± 0.14	0.0005 ± 0.0004	0.16	− 0.10
	Loja	5	7	2	0.40 ± 0.23	0.0019 ± 0.0014	− 1.14	3.36
	Zamora	5	1	2	0.60 ± 0.17	0.0004 ± 0.0004	0.00	0.62

N number of samples, S segregating sites, H number of haplotypes, *H*_*d*_ haplotype diversity, π nucleotide diversity, sd standard deviation. No statistical significance was found in any Tajima’s D or Fu’s Fs values.

Only when COXII and HVRI sequences (1434 bp) were combined was genetic diversity evident within the different groups, and the AMOVA assigned a minor, although significant, component of variability to the populations (42.3%). Haplotype diversity appears to be unchanged within populations when compared to analysis of these sequences using only HVRI (Table 2), but nucleotide diversity decreases as these longer sequences reflect fewer changes. Analysis using Tajima’s D and Fu’s FS indexes did not indicate signals of population expansions or bottlenecks. Interestingly, when these two regions are combined, the Loja UTPL32 sample that originally clustered with the HTOQ3 haplotype found mostly in Quito now clusters independently (Fig. 1c and d).

### SSR amplification, genetic diversity, and population structure analysis

A set of ten microsatellite markers was successfully designed and validated for this species. From the 74 samples, amplification of microsatellite markers was successful for only 36 (22 out of 36 from Quito, 7 out of 26 from Loja Province, and 7 out of 12 from Zamora Province). The Linkage-Disequilibrium analysis with Bonferroni correction for multiple comparisons revealed no association between markers. So, the final dataset included genotypes of 36 individuals with 10 SSR markers with missing data below 5%.

We found a moderate genetic diversity in the analyzed populations (Table 3) with an overall expected heterozygosity (He) of 0.60 and an average number of 5.0 alleles (Na) per locus for Ecuadorian bears. We also found moderate genetic diversity for each of the three populations with similar He values ranging from 0.53 to 0.64. The highest number of private alleles (Pa) was found in Quito (Pa = 13), even when correcting for rarefaction considering uneven sampling (Pa richness corrected 0.63, Table 3). Southern populations had fewer private alleles (Loja Province 4—Pa richness corrected = 0.29, Zamora Province 6—Pa richness corrected = 0.57).

**Table 3:** Genetic diversity and differentiation indexes obtained from SSR analysis.

Population	N	Na	Pa	Ar	He	Ho	Fst	Fis	HWE	IAM	TPM	SSM
Overall	36	50 (5.0)	–	–	0.60	0.51	0.14	0.16	–	0.001	0.101	0.365
Quito	22	40 (4.0)	13 (0.63)	3.04 (2.32)	0.53	0.39	0.25	0.26	0.00	0.492	0.556	0.083
Loja	7	30 (3.0)	4 (0.29)	2.83 (2.47)	0.63	0.55	0.17	0.17	0.03	0.001	0.020	0.005
Zamora	7	32 (3.2)	6 (0.57)	3.04 (2.58)	0.64	0.60	0.08	0.08	0.00	0.001	0.020	0.005

N number of samples, Na total number of alleles (average number of alleles per locus), Pa private alleles (Pa richness corrected for rarefaction), Ar Allelic richness (corrected for rarefaction), He expected heterozygosity, Ho observed heterozygosity, Fst population specific Fst, Fis population specific Fis, HWE P value for Hardy–Weinberg equilibrium test, IAM P value for bottleneck test under the infinite allele model, TPM P value for bottleneck test under the two-phase 70 model, SSM P value for bottleneck test under the stepwise mutation model.

The AMOVA estimated that 26.28% of the total variance is attributed to the populations. Bears from Quito appear to be particularly isolated from populations in the south with a large Nei genetic distance from Loja and Zamora (D > 0.4; Table 4). This suggests that northern and southern populations are distinct. This inference is backed up by Bayesian analysis, which shows population structure (Fig. 1e, K = 2). Moreover, a couple of individuals from the province of Zamora Chinchipe seem to have a significant contribution from a different lineage (Fig. 1e, K = 3). When analyzing clusters derived from a principal coordinates’ analysis (PCA, representing 28.31% of variability), we observe the same pattern indicating that bears from Quito are different from those in the south. Upon analyzing the first 10 PCA vectors that represent 71.46% of the variability as depicted in the DAPC analysis (Fig. 1f), discernible differences can be observed within the two southern groups. The genetic distance between samples from Loja and Zamora is half of the distance observed between southern and northern populations (D = 0.2, Table 4). Formal discriminant analysis derived from DAPC as cluster identification using successive K-means suggest that the most likely scenario is the existence of two genetic clusters (Fig. S2). Specifically, twenty-one out of twenty-two individuals from Quito are clustered in one group, while the fourteen southern individuals and the remaining bear from Quito (DMQ 7) are clustered in the second group (Fig. S3).

**Table 4:** Nei’s genetic distance between populations.

	Quito	Loja
Loja	0.434	–
Zamora	0.457	0.200

Matrix table of pairwise Nei’s genetic distance between the three populations.

Finally, all three populations deviate from Hardy–Weinberg equilibrium (P < 0.05, Table 3), suggesting that evolutionary forces are driving bear populations. Analysis of the excess or deficiency of heterozygosity attributed to population bottlenecks showed that it is likely that southern populations underwent bottleneck events in the recent past (two-tailed P < 0.05, Table 3).

## Discussion

### Genetic diversity and population structure inferred from mitochondrial DNA

We previously reported that the bear population from Quito displayed remarkably low genetic diversity when analyzing the HVR1 of the mtDNA^34^. Here we found even lower haplotype/gene diversity in the populations from southern Ecuador (Tables 1, 2). These results are reflected in the low variability we found in the mitochondrial DNA in this species, which translates into a scarce number of polymorphic sites (Table 1). Ruiz-Garcia et al. suggested that the low genetic diversity reported from the HVR1 in a previous preliminary study of Ecuadorian bears^34^ is misinterpreted due to the reduced number of samples from a constrained geographic area^29^. Here we show that the overall genetic diversity is still low when expanding the sampling to include more samples from very distinct geographical regions (Table 1), or an additional long mtDNA gene (COXII), especially when we compare the diversity indexes with other studies that also analyzed the HVR1 of the D-loop from very small geographic areas in other bear species^35,36^. We acknowledge that the interpretation of genetic diversity indexes of the mitochondrial DNA in *Tremarctos ornatus* and their comparison with indexes of other bear species is problematic due to the recent radiation of the Andean bear across its distribution^17,29^. Alternatively, merging the two southern populations might potentially increase the Hd values. However, haplotype distribution and phylogenetic analysis of the sequences show that the southern populations exhibit distinct differences in mtDNA analysis. Hence, we decided to analyze Loja and Zamora separately.

Our results on haplotype distributions and the high prevalence of exclusive haplotypes contrast with the recent results obtained by Ruiz-Garcia and collaborators where there was no population structure alongside the Ecuadorian Andes^28^. We must point out that our sampling was more exhaustive in these three localities compared to the mentioned study (Zamora Chinchipe 12 samples vs 5; Loja 26 samples vs 5, and Quito 36 samples vs 10). Our analysis of the two mtDNA regions (D-loop and COXII) could have been compared to sequences from previous studies in Ecuador^29^. However, we were unable to make comparisons because sequences from these studies were not publicly available.

When analyzing the HVR1 alone, there is the possibility that the shared haplotypes between Quito and Loja (HTOQ1 and HTOQ3, Fig. 1b) belong to a set of ancestral haplotypes, and although the populations might have differentiated, the haplotypes are still present in both because there has not been sufficient time for the appearance and fixation of new mutations this particular region of the mtDNA in these populations. Only when COXII sequences were included, a sample from Loja that previously grouped with HTOQ3, segregated into a distinct haplotype (Fig. 1c and d). This observation could suggest that potentially the other Loja samples that clustered with Quito samples when only the HVR1 region was analyzed could be different from those from Quito if other markers are included.

The pattern of exclusive haplotypes in the three populations (Fig. 1b) and the high molecular variance (particularly when analyzing the COXII gen alone) between them suggest population structure driven by the effects of female philopatry in Andean bears. This is particularly evident as the nuclear markers cluster the two southern populations together, yet there is a clear differentiation between them when analyzing the mtDNA data. Other studies have found that females establish home ranges close to where they were born and stay for a long period in one area while caring for young cubs, while males may disperse much greater distances^37–39^. While data on dispersal distance for this species is still lacking, home range estimate for males is significantly greater than females^40^ supporting our hypothesis that females are indeed subjected to site fidelity. Unfortunately, confirming the sex of most individuals, even in Quito and Zamora where we matched the collected samples to camera trap data, proved challenging. Although the bear population in northwestern Quito is relatively well studied in this regard^41^, information remains limited and most of the individuals are presumed to be males due to their large size in camera trap photos. Future genetic testing would be of importance to determine the sex of individuals and its impact on dispersal patterns. Thus, further analysis of sex-biased dispersal is not possible with the current available information.

### Genetic diversity and population structure inferred from SSR markers, and comparisons with previous studies

We report higher genetic diversity indexes (He = 0.60; Na per locus = 5) than previous studies in Ecuadorian populations (He = 0.4; Na per locus = 4), and even higher than for the species across its range (He = 0.56)^27^. These results challenge our expectations since markers with tetranucleotide motifs were used preferentially for this study rather than previous studies that used dinucleotide repeats. We expected to find fewer alleles, given that tetranucleotide repeats mutate much less frequently than dinucleotide repeats, making the appearance of a new and increasing number of alleles over time less probable^42^.

Our results using species-specific markers contrast with previous studies where low genetic diversity has been reported for Ecuadorian Andean bear populations. Therefore, the low genetic diversity indexes previously recorded for this species in Ecuador and throughout its distribution could be attributed to ascertainment bias, as discussed above^25,29^. It is worth mentioning that microsatellite markers tend to underestimate genetic variability calculations in carnivores, yet they still prove useful for analyzing population structure among populations^43^. This is especially relevant when working with non-invasive samples obtained from different bear species^44^, including the Andean bear, which yields low amounts of DNA that can be amplified with PCR-dependent molecular markers (such as SSR markers). Using non-invasive samples is important because invasive sampling (e.g., blood or tissue) is discouraged to avoid stressing the animals^45^.

It was previously argued that Ecuadorian Andean bears have no spatial genetic structure and that the country’s population should be treated as a single unit for conservation purposes^27,29^. However, our results suggest a structure between northern and southern populations (Fig. 1e). These results are consistent with mtDNA data, as the same structure patterns were found when analyzing mtDNA haplotype distributions (Fig. 1b and c).

In our analysis, we found that one individual from Quito was consistently clustered with southern bears in both the STRUCTURE and the K-means clustering analysis for population assignment. Although this could suggest this individual had dispersed, it is highly unlikely that an Andean bear, in recent decades, would or could have safely dispersed between Loja and Quito. As mentioned above, there is no published information on Andean bear dispersal distances. Even when using dispersal data for male American black bears (*Ursus americanus*) with average distances between approximately 30–60 km and a maximum distance of 251 km^46^, the theory that an individual would have been able to disperse between these two populations in the Ecuadorian landscape is not supported. A dispersing individual would need to pass through more than 400 km of patchy bear habitat that is fragmented by major highways and roads, rural and urban human settlements, and agricultural areas (Fig. 1a). If genes had been passed south to north through breeding of intermediate populations, we would expect more of the Quito individuals to have clustered with the southern populations or have a larger penetrance of the southern lineage in the northern populations in the STRUCTURE analysis. This was not the case, thus our population assignation analysis of individuals shows that there is practically no geneflow or connectivity between these northern and southern populations. So far, few studies have directly compared the use of heterologous and species-specific primers for microsatellite analysis and their impact on population genetics inferences. It has been observed that the number of alleles and the heterozygosity values increase when using species-specific designed markers^47^, which is the case in our study. This is mainly attributed to non-detected alleles (a.k.a. null alleles) due to mutations in the primer binding sites. Therefore, there is a loss of information when using nonspecific primers^47,48^. This impacts genetic diversity estimates, yet the degree to which this bias leads to mistaken conclusions regarding population structure is still an open question^47^.

We observed smeared peak patterns typical for dinucleotide markers in Tor10, designed to target a dinucleotide locus, and in Tor5 and Tor13, which were imperfect microsatellites resulting in a mix of 4 + 2 nucleotide motifs (Fig. S4). We later removed Tor13 due to having a large and significant null-allele estimation (Table S1). So, it is probable that the smeared pattern raised the difficulty of assigning genotypes unequivocally in these loci and accounted for some miss-genotyping bias^32,33^ which was reflected in null alleles estimation due to modification in the overall allele frequencies. Therefore, here we observe that even with specifically designed markers, smeared patterns in dinucleotide markers might introduce genotyping bias as previously suggested^32,33^. This bias is exacerbated when using heterologous primers, which has been the case for previous Andean bear studies^25–27,29^. When assembling Andean bear SSR loci, we observed mutations in the primer binding sites of the dinucleotide loci previously used for Andean bear studies (Fig. S5). Due to this evidence, we decided to design a different set of specific primers for the study of SSR loci in the Andean bear.

### Implications on conservation planning for Andean bear and biodiversity hotspots

Our findings are particularly relevant for conservation because: (1) it appears that Andean bear populations in Ecuador, and potentially across their distribution, have a higher genetic diversity than was previously thought, and (2) we found evidence that bear populations from different geographic areas in Ecuador are genetically distinct. Our results suggest population structure between northern and southern populations, which was not unexpected for populations so far apart and separated by both rugged topography and centuries of human activities in the Andes. We were, however, surprised by the genetic differentiation found between the two southern sampling sites (as suggested by the mtDNA data in Loja and Zamora Chinchipe Provinces) due to their geographic proximity (Euclidian distance =  < 100 km). As has been found in other bear species^7,49,50^, these results may indicate that fragmentation of this landscape is related to human activities (e.g., deforestation, agriculture, roads, and extractive activities) and their influence on bear population dynamics that could be significantly affecting gene flow between populations within relative proximity. It is also likely that the selective foraging behavior of Andean bears influenced by microtopography contributes to the establishment of discrete populations^51^ as the Andean Depression of southern Ecuador exhibits unique phytogeographic features partly due to the barrier effects of the last glaciation^52^. Whether population structure is attributed exclusively to human activity or (phyto)geography is not clear. The most likely scenario is that both factors could be contributing to the differentiation observed in these populations. Andean bears are among the least studied bear species and there is a lack of historical data on population distribution (i.e. museum samples). For decades, Ecuador has had the highest deforestation rate in South America^53,54^, and the currently Ecuadorian Andes are severely fragmented mainly due to road construction and the use of the land for agriculture (Fig. 1a). Further genomic analyses could help to gain a more precise understanding of these populations and the species history as has been performed for severely fragmented populations in other bear species^55^.

Ruiz-García and collaborators^29^ found no significant distinction between the Andean bear populations in Ecuador and suggested they all be treated as one management unit. Our results indicate this is not the case, and they should be managed separately. The lower genetic diversity found in the northern population (as shown by SSRs) may further indicate the need for specific conservation actions targeting this population which is severely fragmented and surrounded by agricultural land and large human settlements (Fig. 1a). Additionally, the poor connectivity between southern populations (as seen in the mtDNA analysis) indicates the need for further research on gene flow barriers and the implementation of specific conservation activities.

Our samples were collected within or adjacent to two of the four areas identified as conservation nuclei for this species in Ecuador and suggested as viable areas for landscape-scale conservation of an ecologically functional population^21^. The samples from Loja Province are also from the newly designated Sangay-Podocarpus connectivity corridor^56^ (Fig. S6). The samples from Zamora Chinchipe Province in the Cordillera del Condor, a small mountain chain with high biogeographic interest comprising tepui formations and high endemism, are included within the Tropical Andes biodiversity hotspot^57^. Although the sample size of these southern populations is smaller than the sample size from Quito, it is important to note that Andean bears are difficult to study due to their elusive behavior and lower density estimates when compared to other bear species^17,41,58,59^. Thus, these small sample sizes still provide valuable information of the status of those populations.

The samples from the Metropolitan District of Quito were collected from the Tumbes-Chocó-Magdalena biodiversity hotspot^57^ (Fig. S6) a region containing the UNESCO designated Chocó Andino de Pichincha Biosphere Reserve and the Andean Bear Corridor^60^. These hotspots are designated as conservation priorities because they have among the highest endemism of plants, birds, amphibians, reptiles, and mammals on earth and are highly threatened^61,62^. Despite these national and global designations, all three sampling areas face common threats from mining, deforestation, roads, urban expansion, and human-wildlife conflict^41,63^. Ecuador has had the highest deforestation rate in South America since the mid-1990s^53^ (Fig. S6). While most intense in the lowlands, montane ecosystems have also experienced high levels of habitat destruction, including the upper montane forests and paramo, which have incredibly high levels of endemism and biodiversity^18,64^. Thus, efforts to conserve Andean bears would also support the conservation of these important areas of high biodiversity and endemism.

## Conclusions

We designed, standardized, and validated ten specific microsatellite markers to analyze non-invasive samples from Andean bears, providing a cheap and easy-to-use tool for studying the genetic diversity, structure, and connectivity of this emblematic species. This is relevant because Andean bear distribution is in countries where resources for biodiversity monitoring and wildlife conservation are especially scarce. Our results demonstrate the importance of species-specific marker development to analyze genetic diversity and how incorrect marker choice can result in a misunderstanding of the genetic structure of wildlife populations, which could lead to the misdirection of conservation efforts. While our results show a higher genetic diversity than previously known with microsatellite data^26,27,29,31^, the threats to Andean bears in Ecuador remain intense and unlikely to be reduced without direct interventions. Additionally, our data indicate that even bear populations in geographic proximity could be significantly separated by intense topography and human activities. Threats to Andean bears in Ecuador and elsewhere are likely to increase as human use of their habitat continues, and agriculture and extractive activities drive deforestation, habitat degradation, and bear-livestock conflicts^22,65,66^. Our advancement in molecular marker development and use for this species has important implications for direct spatial conservation planning, which occurs in vastly biodiverse Andean ecosystems^61,67^. Connectivity impacts the maintenance of genetic diversity and dissemination of new allelic variants increasing this species’ ability to overcome environmental challenges and evolutionary success in the long term^68^. For this species to persist in Ecuador, conservation efforts must continue to address threats to bears and find sustainable solutions to conflicts or alternatives for local people.

## Methods

### Study area, sampling, and DNA extraction

We obtained non-invasive samples from 74 wild Andean bears from 2014 to 2020. These included 26 fecal samples from Loja Province in southern Ecuador (2014–2015), hair samples from 12 individuals from the Cordillera del Condor on the southwestern border with Peru, in the province of Zamora Chinchipe (2020), and 36 hair samples previously collected in the Metropolitan District of Quito in the northern region of the country (2016–2018) (Fig. 1a). Hair samples in Quito and Zamora Chinchipe Province were obtained using barbed-wire corrals with a vanilla essence scent lure, as previously described^34,69^, and individual identification was based on camera trap photos of each bear’s unique facial features. Sample collection was conducted according to Ecuadorian legislation with the corresponding permits (MAE-DNB-CM-2015-014, MAE-DNB-CM-2019-0118, MAE-DNB-CM-2015-0016) and following local ethical guidelines to avoid animal stress^45^. DNA was extracted using the DNeasy Blood and Tissue Kit (Qiagen, Hilden, Germany) according to the manufacturer’s instructions with a few modifications. We incubated the samples at 56 °C with protease K overnight, and the final DNA elution was performed 3 times in 30 µL for a total of 90 µL. The final 90 µL were passed through the column twice to improve DNA concentration and yield.

### Mitochondrial D-loop amplification, sequencing, and data analysis

A 612 bp product corresponding to the hypervariable region 1 (HVR1) of the D-loop from the mitochondrial DNA was obtained using the primers Tormt2F: 5′-TAGCTCCACCATCAACACCC-3′ and Tormt2R: 5′-ACTGCGACGAGACCTTTACG-3′ specifically developed for studies in Andean bears^34^. PCR products were sequenced in both directions in an ABI 3730XLs sequencer (Macrogen, Inc., Seoul, South Korea). Consensus sequences of both DNA strands were obtained using PreGap4 and Gap4 from the Staden package^70^. These sequences were aligned and trimmed to equal lengths in MEGA v.11^71^. Haplotype (H) and nucleotide (π) diversity indexes calculations, Fu’s FS^72^ and Tajima’s D^73^ neutrality tests for population expansions or bottlenecks, and the analysis of molecular variance (AMOVA) were performed using Arlequin 3.5^74^. A statistical parsimony haplotype network^75^ was obtained using R v4.2.1^76^ through the function *haploNet* as implemented in pegas^77^. Three homologous sequences available in the NCBI nucleotide database (Accessions: NC009969.1^78^, FM177764.1^79^, MW556430.1^80^) were included in the haplotype network and subsequent phylogenetic analysis.

Finally, phylogenetic relationships between all sampled individuals were reconstructed using a Maximum-Likelihood method^81^ with a Kimura-2 parameter as the substitution model^82^ which was selected as the best using IQ-TREE^83^ (http://www.iqtree.org). Five hundred standard bootstraps were used, and the final tree was displayed using FigTree V1.4.4.

### Microsatellite marker design

We used raw data from two Andean bear genomes (SRA accession numbers: ERX1025773 and ERX1025774)^84^ to successfully map four hundred microsatellite loci reported for other bear species within the Andean bear genomes with the NCBI nucleotide blast tool^85^ using the accession numbers to guide the search. We found 72 loci with a repetition motif other than di-nucleotide, and only 25 of these loci had a good sequencing depth and coverage for assembly in both genomes. Tetranucleotide motif markers were preferentially chosen due to better allele score accuracy than dinucleotide markers^32,33^. The assembly of the microsatellite loci and their flanking regions for each genome was performed using CodonCode Aligner v9.0 (CodonCode Corporation, Dedham, MA, USA). The assembled sequences were aligned in MEGA v11^71^, and conserved regions were targeted for primer design using the Primer 3 online tool v4.1.0^86^ according to the following parameters: a primer length between 18 and 22 nucleotides, a GC content between 42 to 55%, a melting temperature between 55 and 65 °C, and a product size between 200 and 400 base pairs. Off-targets were assessed using the Primer-BLAST online tool^87^, and the primer set with the least off-target hits was selected. Finally, mFold^88^ and MFEPrimer-3.0^89^ were used to check for possible primer dimers and hairpins before and after attaching the Tail A sequence (5′-GCCTCCCTCGCGCCA-3′) to the 5′ of the forward primer sequences. The Tail A sequence was added as a more resource-efficient approach to fluorescent label the PCR products using a separately labeled primer in the PCR mix as previously described^90^. Thirteen pairs of primers were successfully designed and synthesized for their validation.

### Microsatellite amplification

Twelve markers were amplified without non-specific products (Figs. S7–S9) of which eleven yielded allele peaks (Fig. S4) in our preliminary tests. For final validation, a PCR product of each marker was sequenced in both directions using the deoxynucleotide Sanger technique (ABI 3730XLs) to verify that microsatellite loci were indeed being targeted (NCBI nucleotide database accessions OQ175001-OQ175011). We used these eleven makers for the analysis of Ecuadorian Andean bear populations.

Each locus was amplified independently in a T100 thermal cycler (Bio-Rad Laboratories, Hercules, CA) in a final volume of 30 µL. The PCR reactions were set as follows: 1 unit of Platinum Taq DNA polymerase (Invitrogen, Waltho, MA), PCR buffer 1×, 1.5 mM MgCl_2_, 0.2 mM dNTPs (Invitrogen, Waltho, MA), 0.5 pM reverse primer, 0.2 pM forward primer, 0.5 pM tail-A primer labeled with either VIC, 6-FAM, PET or NED dyes, and 5 ng of DNA. One dye was assigned to each marker and maintained throughout the study. The cycling temperature profile was set as follows for all the markers: initial denaturation at 94 °C for 7 min, 40 cycles of denaturation at 94 °C for 30 s, annealing at 60 °C for 30 s, extension at 72 °C for 30 s, and a final extension at 72 °C for 5 min. PCR products were resolved through 2% agarose gel electrophoresis with Syber Safe staining (Invitrogen, Carlsbad, CA). Only twelve of the thirteen markers were amplified successfully under these conditions, and eleven markers yielded clear peaks. So, we genotyped our samples with these eleven markers (Table S1).

In a few cases, samples with very low quality and quantity of DNA, especially fecal samples, were hard to amplify. We performed a two-step approach to amplify the specific loci and label the PCR product. If a marker did not amplify, a PCR was performed as previously described with the following modifications. The final volume of the reaction was reduced to 10 µL, MgCl_2_ was raised to 2 mM, the primer concentration of the forward primer was raised to 0.5 mM to match the reverse primer concentration, the Tail-A labeled primer was removed from the reaction, BSA was added in a concentration of 0.02 µg/µL, and the number of cycles was incremented to 55 as previously described for the Andean bear microsatellite amplification^25^. The amplifications were checked on agarose gel electrophoresis, and if the desired product was present, a second PCR reaction was carried out in 30 µL as follows: 1 unit of Platinum Taq DNA polymerase (Invitrogen, Waltho, MA), PCR buffer 1×, 1.5 mM MgCl_2_, 0.2 mM dNTPs, 0.5 pM reverse primer, 0.5 pM of labeled tail-A primer, and 1 µL of the first PCR product. The cycling temperature profile was set as follows: initial denaturation at 94 °C for 2 min, 35 cycles of denaturation at 94 °C for 15 s, annealing and extension at 65 °C for 30 s, and a final extension at 72 °C for 5 min. PCR products were resolved in 2% agarose electrophoresis.

### Microsatellite data analysis

After samples were amplified and dye tagged, we stored them at − 20 °C protected from light, until samples could be sent for analysis. Up to 4 PCR products with different dyes were pooled, mixing 10 µL of each in a plate well. The 96-well plates were sent for capillary electrophoresis in an ABI 3730XL using the GeneScan™ 500 LIZ™ (Life Technologies, Woolston, UK) as the fragment size standard.

Geneious Prime v2022.2.1^91^ was used to perform the allele size call and binning. Coancestry v1.0.1.11 was used to obtain paired relatedness indexes in dyads of individuals to identify closely related individual pairs and remove samples from the analysis if necessary. We used R v4.2.1^76^ and implemented a pipeline for data analysis. We deposited our R script online (see data availability) which also contains a detailed description of each test to allow future comparative and replication studies. A Linkage-Disequilibrium analysis (genepop^92^) was performed to evaluate the independence of the markers. Null-alleles (PopGenReport^93,94^) frequencies were obtained and the data from Tor13 was removed from the analysis as this marker contained the largest null-alleles frequencies. Expected heterozygosity (He), observed heterozygosity (Ho), the number of alleles (Na) (polysat^95,96^ and diveRsity^97^), private alleles (Pa) (proppr^98,99^), allelic richness (Ar) (pegas^77^), Nei’s pairwise genetic distances^100^ (adegenet^101,102^), population-specific Fst and Fis^103^ (hierfstat^104^), AMOVA (pegas^77^), a principal coordinates analysis (PCoA) (ade4^105–107^), a discriminant analysis of the principal coordinates (DAPC)^108^ and cluster identification using K-means (adegenet^101,102^) were obtained from the microsatellite markers data using our R pipeline. Additionally, HP-Rare v1.0^109^ was used to calculate allele and private allele richness corrected by rarefaction due to the uneven sampling size of the dataset. Bottleneck v1.2.02^110^ was employed to evaluate the possibility of the populations having experienced recent bottleneck events under the infinite allele model IAM, the stepwise model SSM, and the two-phase model TPM 70. Finally, using Bayesian inference, Structure v2.3.4^111^ was employed to analyze the genetic structure among northern and southern Ecuador populations. One million Markov chain Monte Carlo steps were run after one hundred thousand burn-in steps under the admixture model. The admixture model was selected under the assumption that there is some degree of gene flow in discrete populations^112^ as suggested in previous Andean bear studies (Ruiz-Garcia^20^). Runs were performed 10 times for each K from 1 to 10. The optimal number of genetic clusters (K) was obtained through the Puechmaille method^113^ which can recover the correct population structure when sampling information is uneven using MaxMeanK, MedMeanK, MaxMedK, MedMedK, and through the widely used Evanno method^114^ which uses and ad hoc approach to calculate the rate of change in the log probability of data between successive K values. The consensus admixture plot from the 10 independent runs was obtained with the CLUMPAK tool as implemented in the online tool Structure Selector^115^. The allelic matrix for our study is deposited online (see data availability) for future comparisons with other bear populations.

### Mitochondrial COXII primer design, sequencing, and data analysis

Three complete annotated mitogenomes of the Andean bear were retrieved from the NCBI nucleotide database (Accessions: NC009969.1^78^, FM177764.1^79^, MW556430.1^80^). Two other mitochondrial DNA genomes were assembled from raw data from short-read sequences (SRA accession numbers: ERX1025773 and ERX1025774)^84^ and aligned to identify conserved regions flanking variable mitochondrial genes for primer design. We identified the whole cytochrome oxidase II (COXII) gene as being one of the longest and most variable among the coding regions of the mitochondrial DNA. We decided to design a different primer set to amplify a longer fragment compared to the one used by Ruiz-García and collaborators^29,116^ and that was specific for the Andean bear as we found mismatches in the primer binding sites when we ran a primer-BLAST analysis targeting *T. ornatus* accessions. The primer design parameters and *in-silico* validation were performed as previously described for SSR markers with the target of a product over 1000 bp flanking the whole COXII gene and extending for a few nucleotides into the flanking COX1 and ATP8 genes. We successfully designed and validated the primer set Tor_COXII_F: GATGCCCTCCTCCGTATCAC and Tor_COXII_R: GGTGGAAAAGGTTTTAGTTCGGG, which yielded a 1082 bp product (vs 783 product from Ruiz-Garcia^20^). PCR amplifications were conducted as it follows: A final volume of 25 µL, 1 U of Taq Platinum DNA polymerase (Invitrogen, Waltho, MA), Buffer 1×, MgCl_2_ 1.5 mM, dNTPs 0.2 mM, 0.25 mg/mL BSA, 0.5 pM of each primer and 2 ng of DNA. The temperature cycling profile was conducted as following: initial denaturation 94 °C for 5 min, followed by 35 cycles of denaturation at 94 °C for 30 s, annealing at 60 °C for 60 s, extension at 72 °C for 1 min, and a final extension at 72 °C for 5 min. PCR products were revealed with an 1.5% agarose gel electrophoresis with SyberSafe staining.

Nineteen samples (9 from Quito, 5 from Loja, and 7 from Zamora) were selected for amplification. This sample set was diverse and represented nearly all identified haplotypes found within the HVRI region. A reduced sample set was used this time, as we had to select samples from which we still had sufficient and good quality DNA to continue with the assays. Data analysis for these samples was performed individually using the COXII sequences as well as merging them with the HVRI sequences following the methods previously described in “Mitochondrial D-loop amplification, sequencing, and data analysis” section.

### Supplementary Information

Supplementary Information.

## Data Availability

We have deposited our SSR allelic matrix in genepop, our mtDNA sequences from the D-loop in Fasta format, alignments mentioned in Fig. S5, and our Rscript for microsatellite data analysis on 10.6084/m9.figshare.20740132.
