# Supplementary material for: Evidence of population genetic structure in Ecuadorian Andean bears
Source: Sci Rep. 2024 Feb 3;14:2834. doi: 10.1038/s41598-024-53003-5 (PMC10838292; doi:10.1038/s41598-024-53003-5)
Supplement: Supplementary file 1 — Supplementary Information. [file 41598_2024_53003_MOESM1_ESM.pdf]

Evidence of population genetic structure in Ecuadorian Andean bears

SUPPLEMENTARY INFORMATION

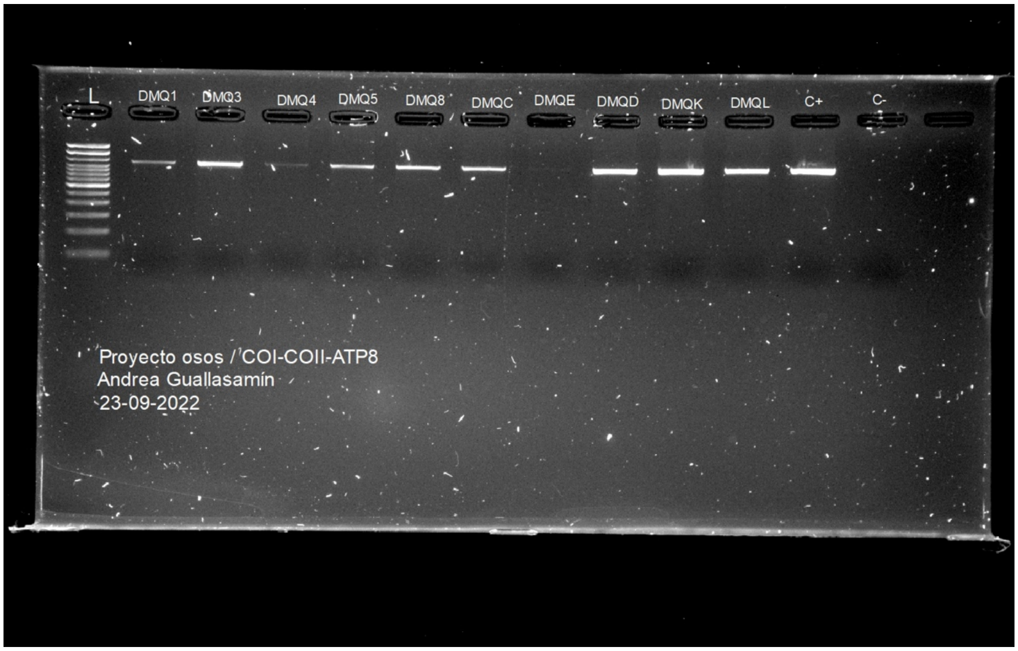

Figure S1: Agarose gel electrophoresis (2%) of the COXII mtDNA gene. A size marker of 100bp was used.

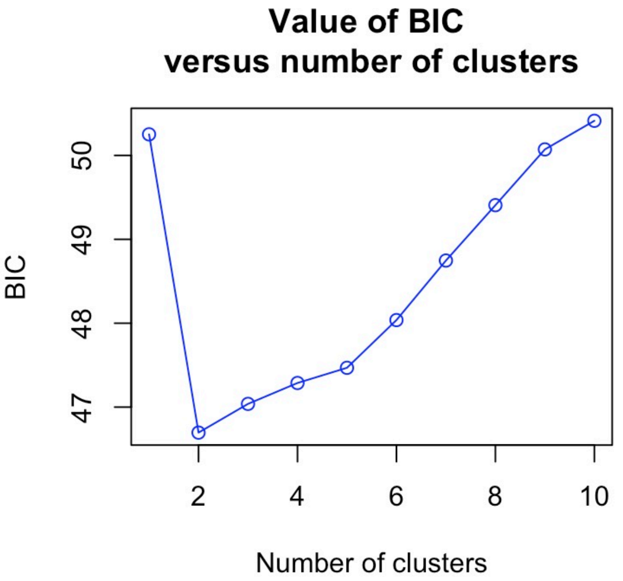

Figure S2: BIC criterion for estimating the number of clusters for the population assignation of Andean bear individuals using SSR markers. The existence of two clusters is the most likely scenario because it had the lowest BIC value.

```
$Kstat
     K=1      K=2      K=3      K=4      K=5      K=6      K=7      K=8      K=9     K=10
50.25121 46.69609 47.03849 47.28754 47.46866 48.03738 48.74709 49.40698 50.07171 50.41290

$stat
     K=2
46.69609

$grp
 DMQ1  DMQ2  DMQ3  DMQ7  DMQ8 DMQ10 DMQ12 DMQ13 DMQ17 DMQ19 DMQ20 DMQ21 DMQ22 DMQ23 DMQ24 DMQ25  DMQB  DMQC  DMQD  DMQF  DMQI  DMQK    L1    L2    L3    L5    L6
    1     1     1     2     1     1     1     1     1     1     1     1     1     1     1     1     1     1     1     1     1     1     2     2     2     2     2
   L8    L9    Z1    Z7    Z9   Z10   Z12   Z13   Z17
    2     2     2     2     2     2     2     2     2
Levels: 1 2

$size

 1  2
21 15
```

Figure S3: Output of the Andean bear population assignation test. DMQ: Quito, L: Loja, Z: Zamora. Samples from Loja and Zamora were clustered in a separate group from the sample from Quito.

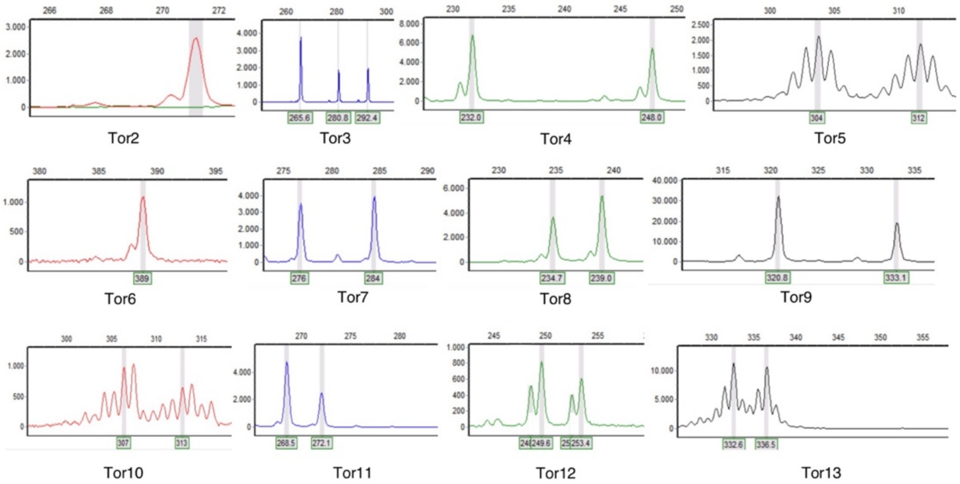

Figure S4: Electropherograms of SSR allele peaks for the twelve originally amplified markers. Tor3 was excluded due to having multiple peaks in all the analyzed samples, and Tor13 was discarded due to having the largest null allele frequency. Thus, the amplification of the final dataset was performed with only 10 markers. Tor5, Tor10, and Tor13 displayed smeared peak patterns typical of dinucleotide SSR markers or imperfect SSR markers with a dinucleotide motif. Individual images of allele peaks were merged into this image to show which peak was considered an allele.

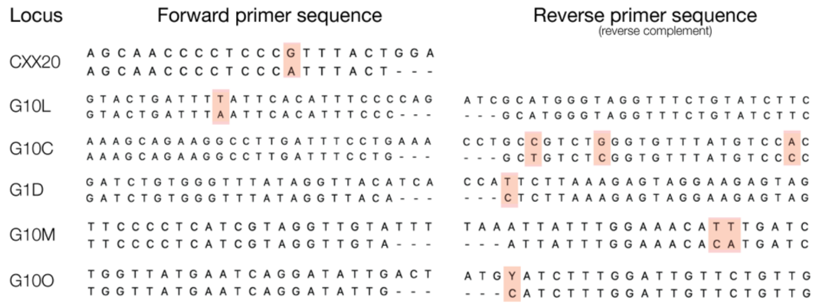

Figure S5: Alignment of primer sequences and primer binding sites of six dinucleotide microsatellite loci used in previous Andean bear genetic studies. We could assemble without gaps only six microsatellite loci used in previous studies. For the locus CXX20, we partially assembled the sequence length; thus, only the forward primer sequence could be aligned with the contig. Mismatches between primers and their binding sites are highlighted in red. We found mismatches in all the analyzed loci, either in the forward or reverse primer binding sequences. These alignments are deposited in our online repository.

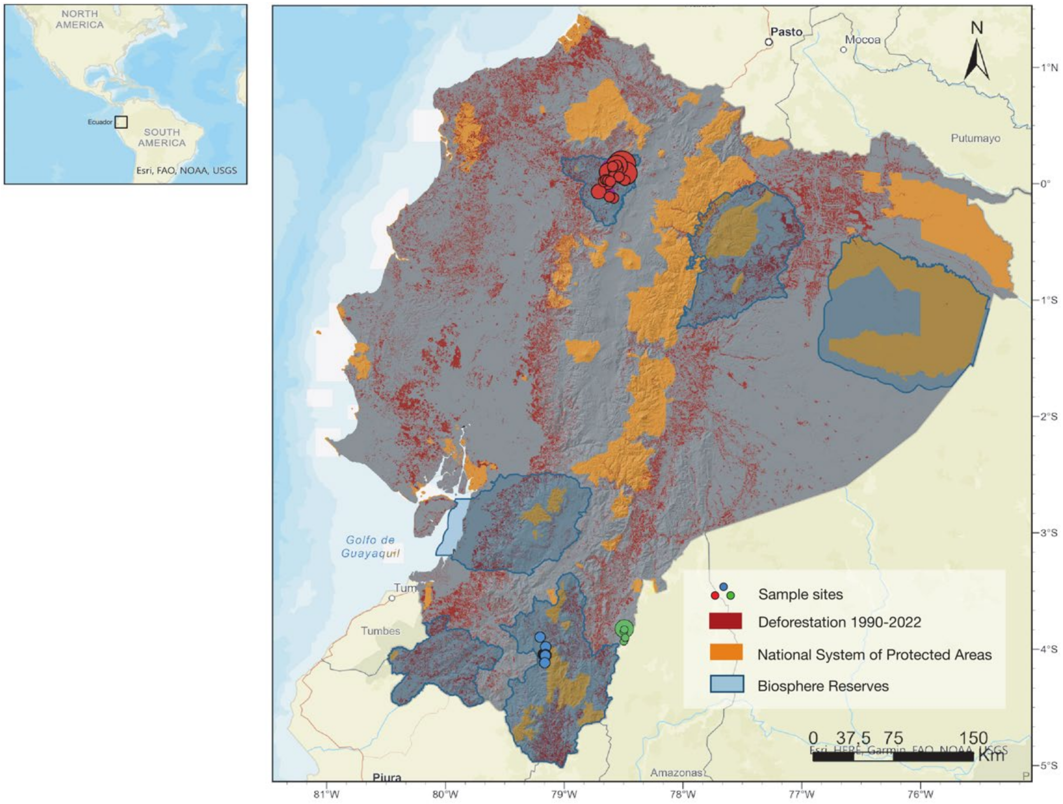

Figure S6: Historical deforestation and protected areas of Ecuador. Layers for deforestation during the las three decades, protected areas and the biosphere reserves are displayed. Map created with ESRI ArcGis Pro v.10.8.2. Map layers source: http://ide.ambiente.gob.ec:8080/mapainteractivo.

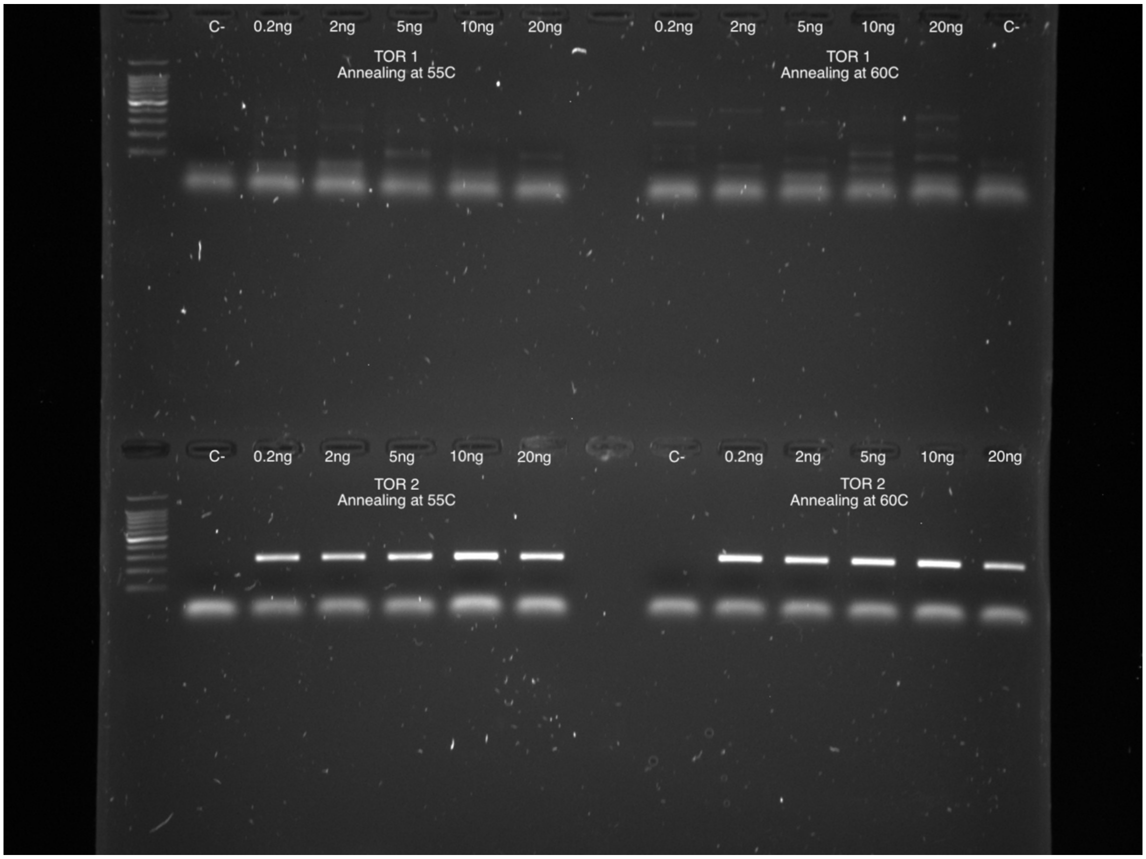

Figure S7: Agarose gel electrophoresis (2%) of the amplification of SSR markers Tor 1 and Tor2 using different DNA concentrations and two annealing temperatures. A 100bp ladder was used as the size standard. The gel was imaged with a Gel Doc XR+ (Bio-Rad Laboratories, Hercules, CA) imaging system at 300 dpi resolution. No modifications have been performed to the gel other than labeling.

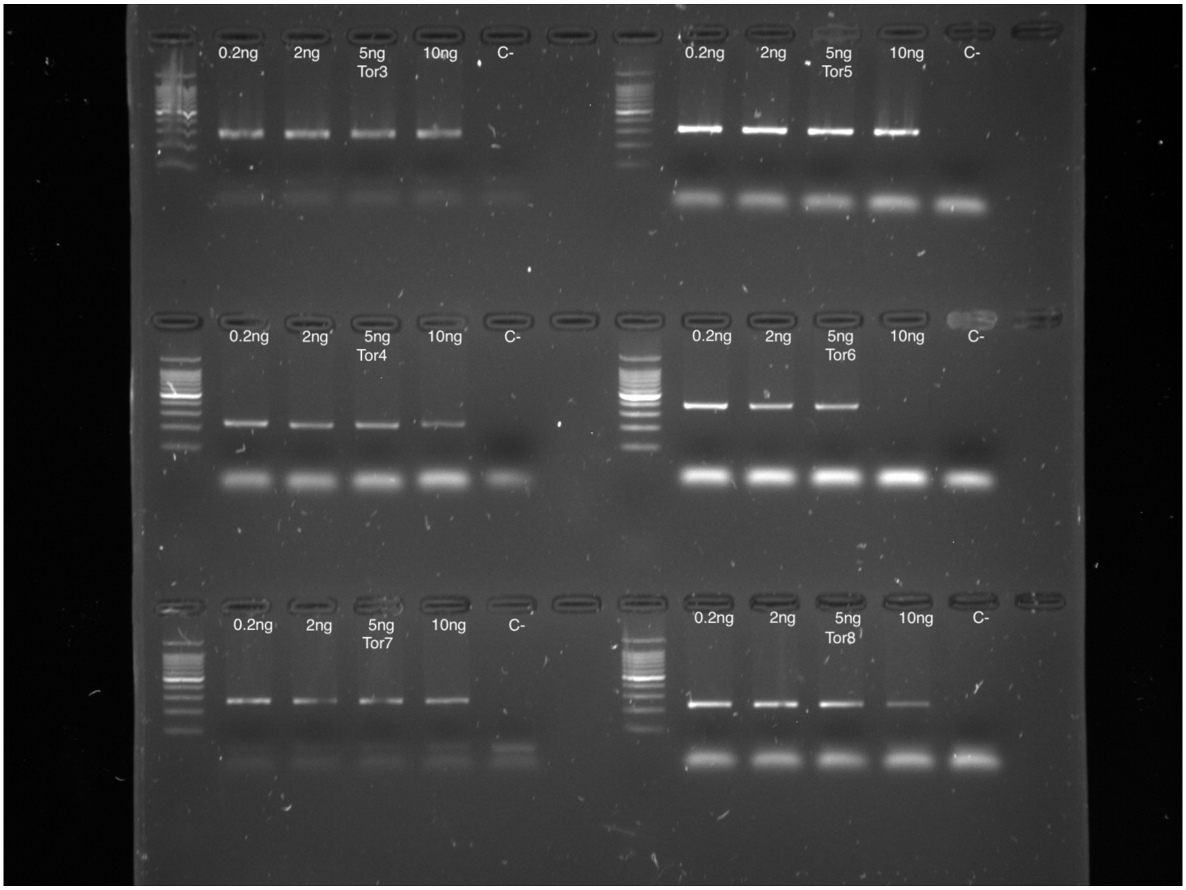

Figure S8: Agarose gel electrophoresis (2%) of Tor 3, Tor4, Tor5, Tor6, Tor7, and Tor8 SSR markers' amplification using different DNA concentrations and an annealing temperature of 60ºC. A 100bp ladder was used as the size standard. The gel was imaged with a Gel Doc XR+ (Bio-Rad Laboratories, Hercules, CA) imaging system at 300 dpi resolution. No modifications have been performed to the gel other than sample labeling.

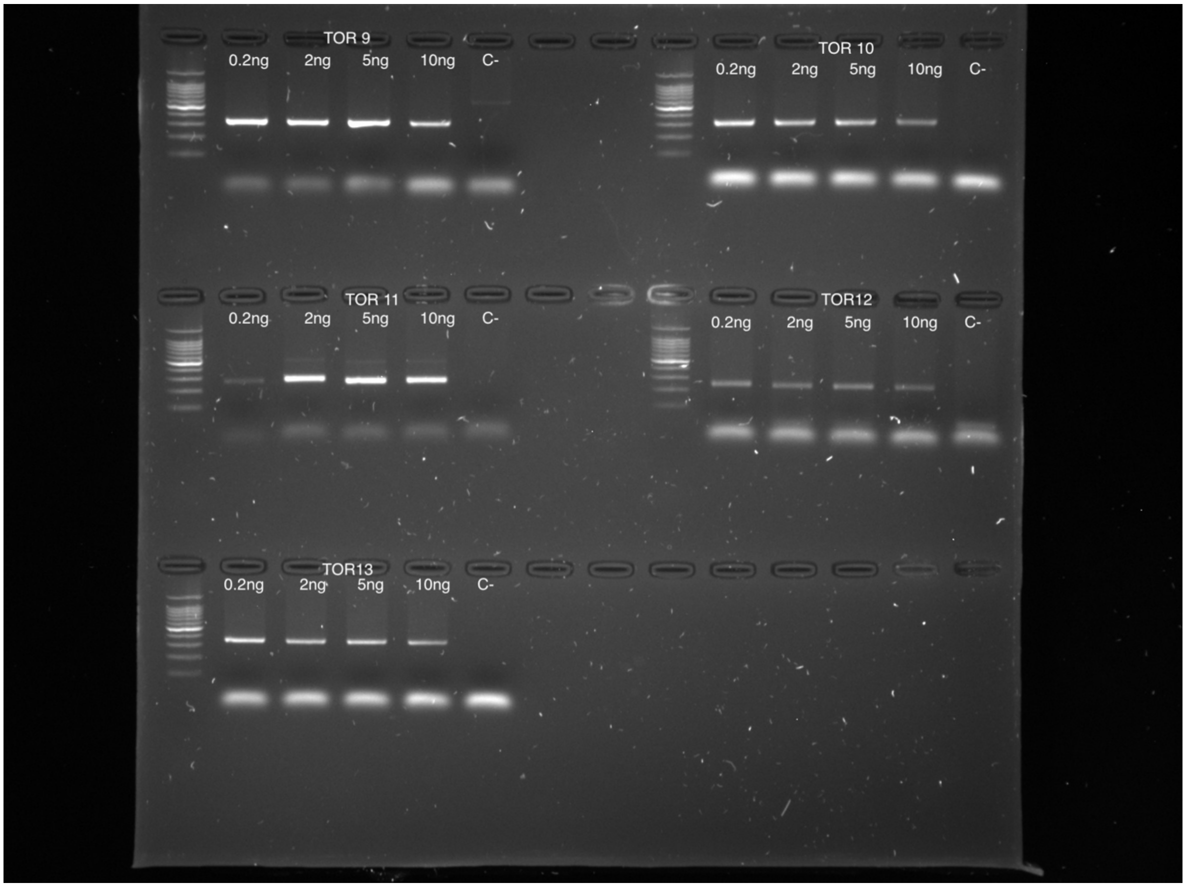

Figure S9: Agarose gel electrophoresis (2%) of Tor9, Tor10, Tor11, Tor12, and Tor13 SSR markers' amplification using different DNA concentrations and an annealing temperature of 60ºC. A 100bp ladder was used as the size standard. The gel was imaged with a Gel Doc XR+ (Bio-Rad Laboratories, Hercules, CA) imaging system at 300 dpi resolution. No modifications have been performed to the gel other than sample labeling.

Marker name	Primer sequence 5'-3'	Repetition motif	Dye in Tail A primer	Alleles size range	Alleles per locus	He	Ho	Observed null allele frequency	NCBI accessions of SSR loci sequences
Tor2	F: *AGAAATGGGCAAAGGACTCAA<br>R: AACAGCAGTGAACAAGGGTTTC	$(cttt)_{10\text{-}13}$	PET	267-279	4	0.46	0.19	0.22	OQ175001
Tor4	F: *CGATGGCACCTATTTGCAG<br>R: TTGGGTTAGGATTACACAGCA	$(aaag)_{12\text{-}17}$	VIC	232-252	6	0.65	0.50	0.10	OQ175002
Tor5	F: *AGCAAACCCAAAGACAGAGG<br>R: CGATGTGGGTCTCTTCAGGT	$(tttc)_{9\text{-}31}(tc)$	NED	304-322	6	0.55	0.55	0.00	OQ175003
Tor6	F: *TTACTTCCCCAAGGCACTGT<br>R: TGTGGCAGTGGATCTATTCAG	$(cttt)_{10\text{-}12}$	PET	373-393	6	0.73	0.45	0.19	OQ175004
Tor7	F: *TCACCCAACACTTACCAGGA<br>R: ACAACTGCACATCCTCACTAA	$(ttta)_{10\text{-}12}$	6-FAM	272-284	4	0.71	0.70	0.01	OQ175005
Tor8	F: *CTGTATTGGAGCTCACGACTT<br>R: TTACAGCATAGCACCTGGATT	$(tatc)_{13\text{-}18}$	VIC	231-239	3	0.56	0.55	0.01	OQ175006
Tor9	F: *GCCTTCCCATTTGCTGTAGA<br>R: GGCACAGGAAAACCTTCTCA	$(atag)_{11\text{-}19}$	NED	321-349	6	0.75	0.55	0.12	OQ175007
Tor10	F: *ATTCGGTGGTCAGGTTTGAG<br>R: TAAATGTTGCTTTGGTCCTG	$(gt)_{18\text{-}23}$	PET	305-315	5	0.65	0.29	0.28	OQ175008
Tor11	F: *TGCTGTGGGTACTTGCTGTG<br>R: CGGCGGAACTGAAGTAGATG	$(aaag)_{13}$	6-FAM	268-280	4	0.48	0.38	0.07	OQ175009
Tor12	F: *GCATAATTGCAGAACCAGAGC<br>R: TGAAGCCTAAGTGCCTGTGG	$(tatc)_{8\text{-}11}$	VIC	238-262	6	0.69	0.46	0.15	OQ175010
+Tor13	F: *CCCAGAACACTGTGAAGCAA<br>R: CCATACAGCCCCTTTGTCAG	$(cttt)_{19}(ct)_5(cttt)_8$	NED	301-363	12	0.86	0.35	0.34	OQ175011

Table S1: SSR marker primer set for the present study. *The Tail A sequence (GCCTCCCTCGCGCCA) was attached to the 5' end of the Forward primers. Tor5 and Tor13 corresponded to imperfect microsatellite motifs combining 2 and 4 repetitions as revealed by peak patterns, allele sizes, and sequences. We synthesized only 4 dyed Tail A primers for the whole study with the PET, VIC, NED, and 6-FAM fluorescent dyes, and used them in our PCR reactions. Values of the allele size range, alleles for locus, expected heterozygosity (He), observed heterozygosity (Ho), and null allele frequencies are displayed for each marker. +Tor 13 was removed from the final analysis.
